# Comprehensive and Comparative Metabolomic Profiling of Wheat, Barley, Oat and Rye Using Gas Chromatography-Mass Spectrometry and Advanced Chemometrics

**DOI:** 10.3390/foods3040569

**Published:** 2014-10-31

**Authors:** Bekzod Khakimov, Birthe Møller Jespersen, Søren Balling Engelsen

**Affiliations:** Department of Food Science, Faculty of Science, University of Copenhagen, Rolighedsvej 30, Frederiksberg C, 1958 Copenhagen, Denmark; E-Mails: bm@food.ku.dk (B.M.J.); se@food.ku.dk (S.B.E.)

**Keywords:** GC-MS, metabolomics, barley, wheat, oat, rye, TMSCN, PARAFAC2

## Abstract

Beyond the main bulk components of cereals such as the polysaccharides and proteins, lower concentration secondary metabolites largely contribute to the nutritional value. This paper outlines a comprehensive protocol for GC-MS metabolomic profiling of phenolics and organic acids in grains, the performance of which is demonstrated through a comparison of the metabolite profiles of the main northern European cereal crops: wheat, barley, oat and rye. Phenolics and organic acids were extracted using acidic hydrolysis, trimethylsilylated using a new method based on trimethylsilyl cyanide and analyzed by GC-MS. In order to extract pure metabolite peaks, the raw chromatographic data were processed by a multi-way decomposition method, Parallel Factor Analysis 2. This approach lead to the semi-quantitative detection of a total of 247 analytes, out of which 89 were identified based on RI and EI-MS library match. The cereal metabolome included 32 phenolics, 30 organic acids, 10 fatty acids, 11 carbohydrates and 6 sterols. The metabolome of the four cereals were compared in detail, including low concentration phenolics and organic acids. Rye and oat displayed higher total concentration of phenolic acids, but ferulic, caffeic and sinapinic acids and their esters were found to be the main phenolics in all four cereals. Compared to the previously reported methods, the outlined protocol provided an efficient and high throughput analysis of the cereal metabolome and the acidic hydrolysis improved the detection of conjugated phenolics.

## 1. Introduction

Cereals such as wheat, barley, rye and oat are amongst the mostly grown agricultural food products worldwide and the most important cereal crops for human consumption in northern Europe. The detailed chemical and functional composition of these crops is defining their use for food and feed as well as their prices. Cereals are the most important study objects in foodomics studies seeking to optimize their health beneficial factors and/or reducing deleterious metabolites. While the gross chemical composition, such as carbohydrates, proteins, dietary fibers and micronutrient contents, are important characteristics of cereal products, recent studies showed that relatively low concentration secondary metabolites such as antioxidant phenolics, organic acids and phytosterols have a significant influence on the health and nutritional values of cereals [1,2]. The beneficial health effects associated with the consumption of cereals have been attributed to dietary fiber content [3] as well as phenolics that possess antioxidant, radical scavenging and cholesterol lowering properties [4,5,6,7]. Whole grain barley intake has proven to decrease the low-density lipoprotein (LDL) cholesterol in an intervention study involving hypercholesterolemic patients [8]. Moreover, phenolic acids were found to be important texturizing agents in cooking-extrusion of cereals [9] and recognized as the main antioxidant constituents of cereals [10].

Quantitative and qualitative analysis of both, secondary and primary metabolites (with molecular weight of up to 1500 Da) of grains are studied within cereal metabolomics. Cereal metabolomics offers an insight into the metabolic fluctuations of cereal cultivars that may reveal effects of genetic modifications as well as of biotic and abiotic stresses [11]. Recent studies have illustrated the power of cereal metabolomics to reveal effects of growth temperature [12], salt stress [13], drought stress [14], and biotic stress [15]. Cereal metabolomics is also a promising approach to reveal biochemical and genetic backgrounds of quality traits and may open new possibilities towards targeted breeding [16,17].

Comprehensive metabolomic profiling of cereals requires a reliable protocol that enables extraction of maximum metabolic information in a high-throughput and reproducible manner. Metabolomics studies performed for uncovering single and/or multiple internal and/or external effects on cereals aim to cover as broad range of metabolites as possible. However, due to the great physico-chemical diversity of cereal metabolites, it is in practice impossible to cover the whole cereal metabolome using a single protocol. The phytochemical composition, including phenolics of wheat [18,19,20], barley [21], oat [22] and rye [23] have been investigated in a number of studies within the HEALTHGRAIN diversity-screening program [24].

This study demonstrates the development of comprehensive GC-MS metabolomics protocol for profiling a broad range of phenolics and organic acids from whole grain flour samples, and applied on wheat, barley, rye and oat. Phenolics of cereals are primarily present in conjugated and bonded forms with carbohydrates, lipids and other cell membrane components that alter their solubility and thus bioavailability [21]. Analysis of phenolic content of cereals is mainly performed by basic hydrolysis of cereal extracts [18], which can only cleave ester bonds and stabilize de-esterification reactions. However, a substantial part of phenolics and other organic acids of cereals are conjugated through glycosidic and/or ether bonds to carbohydrates and other molecules. In contrast to basic hydrolysis, acidic hydrolysis allows the cleavage of not only ester bonds, but also glycosidic and ether bonds at an elevated temperature. The advantages of this approach have been demonstrated in polyphenol analysis of the wheat and rice grains [25,26].

In this study, a standardized, high-throughput and unbiased protocol was developed for GC-MS metabolomic profiling of free and conjugated phenolics and organic acids of whole-grain cereals using hydrochloric acid based hydrolysis followed by trimethylsilyl derivatization. The study demonstrates the first application of a novel trimethylsilylation method based on trimethylsilyl cyanide (TMSCN) for derivatization of cereal metabolites. When compared to other frequently used derivatization methods, the new protocol provides a more unbiased and broad-spectrum derivatization of metabolites and is able to provide reproducible metabolomics profiles of complex biological samples [27]. The obtained raw GC-MS data of cereals were processed by a semi-automated multi-way decomposition method, PARAFAC2 [28]. The PARAFAC2 processing of the raw GC-MS data lead to unambiguous deconvolution of elusive peaks such as, overlapped, retention time shifted and low s/n peaks and enable an automatic estimation of relative concentrations of detected peaks [29,30]. Metabolite extraction and GC-MS analysis of the cereal samples were performed within a bigger study, which involved a larger set of barley samples (manuscript in preparation). The main aim of this study was to demonstrate the performance of the protocol, using new technologies within metabolomics, and to show first results of a comparative application to the four major north European cereals: wheat, barley, rye and oat. To the best of our knowledge, this is the first study illustrating a comprehensive GC-MS profiling of phenolics and organic acids of cereals using exactly the same protocol across different cereals.

## 2. Experimental Section

Whole grain samples of wheat (*Tr. aestivum*, variety Bussard), barley (*H. vulgare*, variety Bomi), rye (*S. cereal*, variety Petkus) and oat (*A. sativa*, variety Sang) were purchased in Sepetember 2012 from the Danish bread cereal producing company Aurion (Hjørring, Denmark). All four cereals were grown under biodynamical conditions in Jutland during the season 2011/12.

### 2.1. Metabolite Extraction and Sample Derivatization

Cereal metabolites were extracted from 50 mg of milled grains that were soaked into 600 μL 85% methanol and vortexed for 20 s at 3000 rpm followed by 20 min incubation at 30 °C using a Thermomixer (Model 5436, Eppendorf, Hamburg, Germany) at 1400 rpm. After 3 min of centrifugation at 16,000× *g*, the supernatant was transferred to a fresh 2 mL Eppendorf tube (Hamburg, Germany) and the remaining flour sample was extracted a second time using the same extraction procedure. Then, the combined extracts were completely dried under nitrogen gas flow at 40 °C and hydrolyzed by using 240 µL of 6 M hydrochloric acid at 96 °C for 1 h by stirring at 1400 rpm. The hydrolyzed extracts were transferred into a fresh 2 mL glass vials and phenolics and organic acids were extracted into diethyl ether. Ether-based extraction of phenolics and organic acids was performed twice, by addition of 800 μL diethyl ether and vortexing for 25 s. The obtained ether fractions were completely dried using nitrogen gas flow and re-solubilized in 200 μL 100% methanol. Aliquots, 90 microliter, of the final extracts were transferred into 200 µL glass inserts and completely dried under nitrogen gas flow, sealed and stored at −20 °C until GC-MS analysis. Each sample was spiked with an internal standard (IS) (5 μL of 0.2 mg mL^−1^ solution of ribitol). In order to avoid any moisture, the samples stored in the freezer were dried under reduced pressure before derivatization. Sample derivatization and injection were fully automated by using a Multi-Purpose Sampler (MPS, GERSTEL, Mülheim, Germany) with DualRait WorkStation integrated to a GC-MS system from Agilent (CA, USA). Each sample was individually derivatized by addition of 40 µL trimethylsilyl cyanide (TMSCN) and incubated for 40 min at 40 °C. Two replicate samples per cereal were analyzed in randomized order and the MPS autosampler allowed a sequential derivatization of all samples in the same manner by keeping the derivatization time constant, throughout the analysis.

### 2.2. GC-MS Data Acquisition

The GC-MS consisted of an Agilent 7890A GC and an Agilent 5975C series MSD. GC separation was performed on a Phenomenex ZB 5MSi column (30 m × 250 μm × 0.25 μm). A derivatized sample volume of 1 µL was injected into a cooled injection system (CIS port) using Solvent Vent mode at the vent pressure of 7 kPa until 0.3 min after injection at the vent flow of 100 mL min^−1^. Detailed information on CIS and MPS parameters are described in Khakimov *et al.* 2013 [27]. Hydrogen was used as carrier gas, at a constant flow rate of 1.2 mL min^−1^, and the initial temperature of CIS was set to 120 °C for 0.3 min followed by heating at 5 °C s^−1^ until reaching 320 °C and then held for 10 min. The GC oven program was as follows: initial temperature 40 °C, equilibration time 3.0 min, heating rate 12.0 °C min^−1^, end temperature 300 °C, hold time 8.0 min and post run time 5 min at 40 °C. Mass spectra were recorded in the range of 50–500 *m*/*z* with a scanning frequency of 3.2 scans s^−1^, and the MS detector was switched off during the 8.5 min of solvent delay time and after 25.5 min of the run time. The transfer line, ion source and quadrupole temperatures were set to 290, 230 and 150 °C, respectively. The mass spectrometer was tuned according to manufacturer’s recommendation by using perfluorotributylamine (PFTBA).

### 2.3. Data Analysis

Initial analysis and visualization of the GC-MS data was performed using ChemStation software (Agilent, Germany). Retention indices of detected metabolites were calculated using the Van den Dool and Kratz equation and retention times of C10-C40 alkanes that were analyzed using the same GC-MS protocol [31]. The raw GC-MS data was imported from netCDF format to .mat files into Matlab® ver. R2012b (8.0.0.783) and data was manually divided into 121 smaller baseline separated intervals in retention time dimension. Each interval was modeled separately by PARAFAC2 as described previously [30]. PARAFAC2 modeled the three-way raw GC-MS data (elution time × mass spectra × samples) without any prior data pre-processing. The PARAFAC2 model outcomes: the elution profiles, which represent the TIC in the raw data, and spectral profiles, which represent the experimental EI-MS of deconvoluted peaks, were used for metabolite identification. The PARAFAC2 resolved mass spectrum of each peak was extracted and compared against NIST05 library (NIST, USA), Golm Metabolite Database [32]. Finally, PARAFAC2 concentration profiles, which represented relative concentrations of detected peaks were extracted and normalized according to the peak area of the internal standard (ribitol). The obtained metabolite table was used for exploring variations of phenolics in cereals and for principal component analysis (PCA) [33] after autoscaling of the data.

## 3. Results and Discussion

### 3.1. GC-MS Metabolomic Profiling and PARAFAC2 Based Data Processing

**Figure 1:**
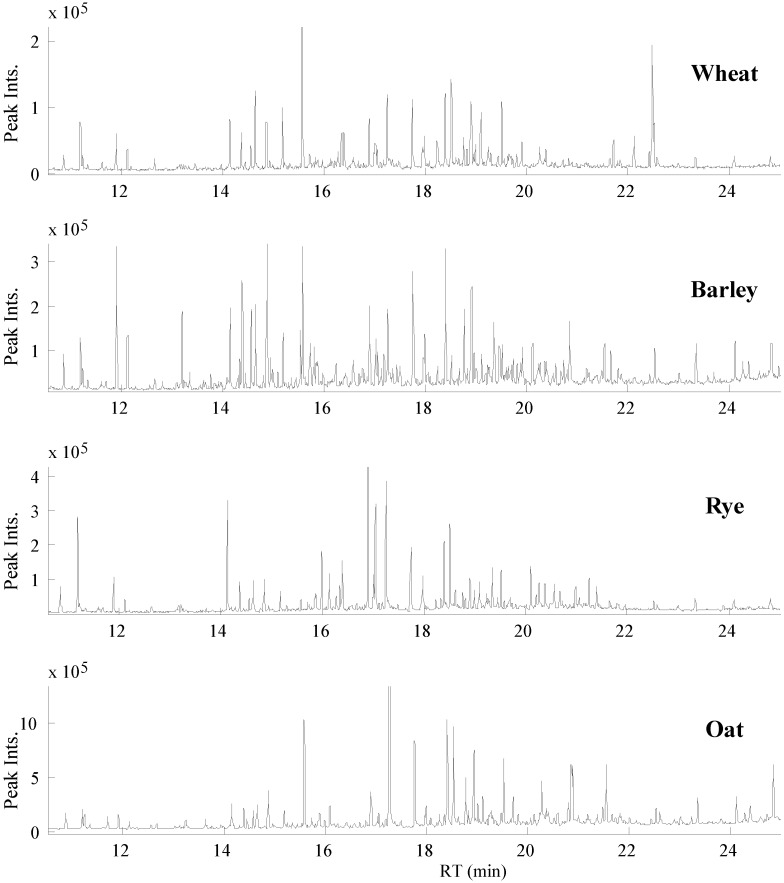
The total ion current (TIC) chromatograms of GC-MS data obtained on wheat, barley, rye and oat metabolite extracts.

**Figure 2:**
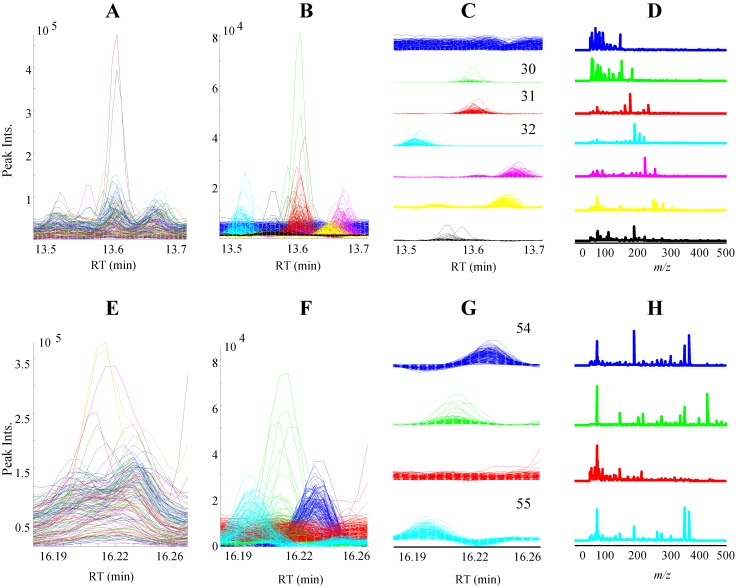
PARAFAC2 based processing of raw GC-MS data intervals. (**A**) and (**E**) are the TIC of raw GC-MS data intervals. (**B**) and (**F**) are the superimposed PARAFAC2 elution profiles of the raw GC-MS data intervals with seven and four components, respectively. (**C**) and (**G**) are subplots of (**B**) and (**F**), respectively. * Numbers of elution profiles correspond to the metabolites represented in Table 1. (**D**) and (**H**) are subplots of PARAFAC2 mass spectral profiles.

The total ion current (TIC) chromatograms of the GC-MS data obtained from hydrolyzed extracts of the four cereals are illustrated in Figure 1. Just over 300 peaks with a s/n ratio >10 were detected from GC-MS profiles. Validated PARAFAC2 models of 121 intervals of the raw GC-MS data revealed 389 components including resolved peaks, shoulders of neighbor peaks and baseline. Then, each PARAFAC2 model was individually evaluated and components that represent baseline, artifact peaks such as column bleed and reagent derived peaks and shoulders of neighbor peaks were eliminated, resulting in 247 chromatographic peaks with unique retention indices and mass spectra. The PARAFAC2 modeling of GC-MS intervals representing vanillin, protocatechuic acid and β-resorcylic acid are demonstrated in Figure 2.

Comparison of RIs and PARAFAC2 resolved mass spectra of 247 resolved peaks against the NIST05 and Golm Metabolite Database resulted in the identification of 89 metabolites (Table 1) at level 2 as described in Metabolomics Standards Initiative report [34]. A total of 32 out of 89 identified metabolites were trimethylsilyl (TMS) derivatives of phenolic acids, their esters and aldehydes. In addition to the previously found phenolic acids from different barley genotypes [21], several other phenolics such as *p*-salicylic, gallic, gentisic, homovanillic and α-resorcylic acids and methyl esters of ferulic, caffeic, protocatechuic and sinapinic acids were identified. Small molecular organic acids, alcohols and their esters constituted 30 out of 89 identified metabolites. These included succinic, glyceric, maleic, fumaric, malic, pyroglutamic, azelaic acids and methyl esters of aconitic and citric acids that are part of the same or different metabolic pathways, and in addition, TMS-derivatives of 10 fatty acids and their esters, 6 sterols and a flavonoid, catechin-*n*TMS.

**Table 1:** A list of identified metabolites from wheat, barley, rye and oat flour samples by GC-MS. Metabolite identification was performed at level 2 as described in Metabolomics Standards Initiative report [34] and was based on RI and EI-MS library match (>80). ^a^ Metabolites with more than one isomers and/or TMS-derivatives; ^b^ tentatively identified.

No	Metabolites	RT min	RI (r)	RI (c)
1.	Laevulic acid-1TMS	9.04	1030	1070
2.	Sorbic acid-1TMS	9.06	1009	1071
3.	Hepta-2,4-dienoic acid, methyl ester	9.28	1000	1080
4.	Octanol-1-1TMS	9.51	1101	1090
5.	Malonic acid-2TMS	9.99	1205	1207
6.	(3,3-Dimethyl-1-cyclohexen-1-yl)oxy]-1TMS	9.97	1110	1206
7.	Benzoic acid-1TMS	10.42	1228	1226
8.	3-Methyl-2-furoic acid-1TMS	10.38	1107	1224
9.	Glycerol-3TMS	10.88	1282	1246
10.	1,3-Dihydroxypropanone-2-2TMS	11.03		1249
11.	Succinic acid-2TMS	11.24	1292	1262
12.	Glyceric acid-3TMS	11.51	1199	1274
13.	Maleic acid-2TMS	11.55	1286	1275
14.	Fumaric acid-2TMS	11.60	1178	1278
15.	*p*-Hydroxybenzaldehyde-1TMS	11.85	1280	1289
16.	2-Hydroxyheptanoic acid-2TMS	11.83	1312	1288
17.	3-Hydroxybutanoic acid-2TMS	12.12	1403	1401
18.	Resorcinol-2TMS	12.2	1378	1404
19.	Trimethyl aconitate	12.50	1428	1419
20.	Citric acid, trimethyl ester	12.82	1442	1435
21.	3-Hydroxyanthranilic acid, methyl ester-1TMS	12.8		1434
22.	2,4-Dihydroxy-5-methylpyrimidine-2TMS	12.89	1403	1439
23.	5-Hydroxy-2-(hydroxymethyl)-4H-pyran-4-one-2TMS	13.08	1492	1448
24.	Maseptol-1TMS	13.12	1358	1450
25.	Malic acid-2TMS	13.19	1494	1453
26.	2-Hydroxycyclohexanecarboxylic acid-2TMS	13.23	1402	1456
27.	3-Hydroxyoctanoic acid-2TMS	13.35	1452	1462
28.	Pyroglutamic acid-2TMS	13.46	1466	1467
29.	Erythritol-4TMS	13.47		1467
30.	Dimethyl azelate	13.61	1485	1474
31.	4-Hydroxybenzeneacetic acid, methyl ester-1TMS	13.62	1458	1475
32.	Vanillin-1TMS	13.55	1469	1471
33.	Citric acid, trimethyl ester-1TMS	13.76		1482
34.	2-Furancarboxylic acid, 5-[(oxy)methyl]-1TMS	13.72	1540	1480
35.	4-Hydroxyphenylethanol-2TMS	13.92	1475	1490
36.	Anozol	14.15	1603	1601
37.	2-Ketoglutaric acid-3TMS	14.34	1622	1612
38.	3-Methyl-3-hydroxypentanedioic acid-3TMS	14.3	1610	1609
39.	Dodecane-6-hydroxy-1TMS	14.40	1631	1615
40.	4-Hydroxybenzoic acid-2TMS	14.45	1618	1618
41.	Methyl Isovanillate-1TMS	14.66	1547	1629
42.	Suberic acid-2TMS	15.11	1682	1654
43.	Syringaldehyde -1TMS	15.15	1658	1656
44.	β-d-Arabinopyranose-4TMS ^a^	15.23	1692	1660
45.	β-d-Xylopyranose-4TMS	15.30	1694	1664
46.	3,5-Dihydroxybenzoic ac. met.est.-2TMS	15.35	1656	1667
47.	2,5-Dimethoxymandelic acid-2TMS	15.38	1867	1669
48.	Vanillic acid-2TMS	15.72	1656	1687
49.	4-Hydroxycinnamic acid, methyl ester -1TMS	15.88	1565	1696
50.	Azelaic acid-2TMS	15.98	1800	1802
51.	2,3-Dihydroxyphosphoric acid, propyl ester-4TMS	15.86	1708	1695
52.	Methyl 2-(oxy)-2-(4-(oxy)phenyl)propanoate-2TMS	16.14	1757	1811
53.	α-d-Galactofuranoside, methyl-2,3,5,6-tetrakis-4TMS ^a^	16.11	1845	1810
54.	3,5-Dihydroxy benzoic ac.-3TMS	16.24	1826	1818
55.	3,4-Dihydroxy benzoic ac.-3TMS	16.20	1826	1815
56.	d-Fructose-5TMS	16.41	1867	1828
57.	Isocitric acid-4TMS	16.34	1835	1823
58.	Catechin-*n*TMS ^a^	16.44		1830
59.	Homovanilic acid-2TMS	16.4	1867	1827
60.	β-d-Galactopyranoside, methyl 2,3,4,6-tetrakis-4TMS ^a^	16.68	1900	1844
61.	Catechin-*n*TMS ^a^	16.77		1849
62.	2,5-Dihydroxy benzoic ac.-3TMS	16.78	1796	1850
63.	α-d-Glucopyranoside, methyl 2,3,4,6-tetrakis-4TMS ^a^	16.90	1928	1857
64.	Syringic acid-2TMS	16.88	1845	1856
65.	β-d-Glucopyranoside, methyl 2,3,4,6-tetrakis-4TMS ^a^	17.05	1928	1866
66.	α-d-Glucopyranose, 1,2,3,4,6-pentakis-5TMS ^a^	17.02	1924	1864
67.	Palmitic acid, methyl ester	17.01	1870	1864
68.	d-Galactose, 2,3,4,5,6-pentakis-5TMS ^a^	17.12	1970	1871
69.	*p*-Coumaric acid-2TMS	17.18	1924	1874
70.	Ferulic acid, methyl ester-1TMS	17.25	1765	1878
71.	3,4,5-Trihydrozy benzoic ac.-4TMS	17.45	1976	1890
72.	2-Hydroxymandelic acid, ethyl ester-2TMS	17.34	1777	1884
73.	4’-Cyclohexylacetophenone	17.58	1703	1898
74.	Caffeic acid methyl ester-2TMS	17.76	1863	2010
75.	β-d-Glucopyranose-5TMS ^a^	17.75	1970	2009
76.	2-Hydroxysebacic acid-3TMS	18.13	2059	2034
77.	Ferulic acid-2TMS	18.40	2076	2052
78.	8,11-Octadecadienoic acid, methyl ester	18.35	2093	2049
79.	Sinapinic acid methyl ester-1TMS	18.51	1943	2059
80.	Methyl vanillactate-2TMS	18.55	2030	2062
81.	Caffeic acid-3TMS	18.76	2114	2076
82.	9-Methoxy-4α-methyl-2,3,7-trihydroxy-4,4a-dihydro-2H-benzo[c]chromen-6(3H)-one ^b^	18.85		2082
83.	Linoleic acid-1TMS	19.23	2202	2207
84.	4,8-Dihydroxy-2-quinolinecarboxylic acid-3TMS	19.46	2265	2224
85.	Sinapinic acid-2TMS	19.52	2221	2228
86.	Androsterone type plant sterol ^b^	19.89		2254
87.	3-Hydroxyandrostan-17-one-1TMS	19.98	2186	2261
88.	19-Norandrosterone-3-TMS ^b^	20.36	2198	2288
89.	9,10-Dihydroxystearic acid-3TMS	20.87	2517	2426
90.	3,7-di-Hydroxy-androstan-17-one-2TMS	21.09	2432	2443
91.	9,10-Dihydroxystearic acid, dimethyl ester-2TMS	21.49	2784	2474
92.	2,3-Dihydroxypalmitic acid, propyl ester-2TMS	21.84	2581	2601
93.	2-Deoxy-6-phosphogluconolactone-5TMS	23.26		2820
94.	2-Hydroxytetracosanoic acid, methyl ester-1TMS	23.69	2894	2858
95.	3,7-Dihydroxycholest-5-ene-2TMS	23.95	2900	2881

### 3.2. Principal Component Analysis (PCA)

**Figure 3:**
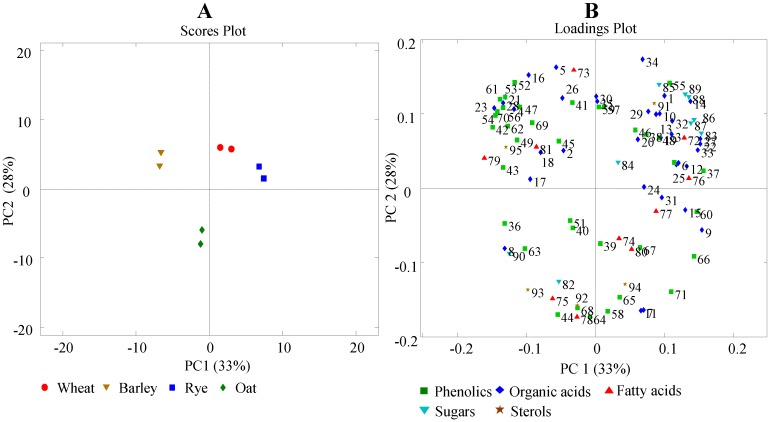
(**A**) scores and (**B**) loading plots of the three component PCA model developed using identified metabolite table. * Numbers in loadings plot correspond to the metabolites represented in Table 1.

In order to explore the metabolomics data, PCA was performed on the metabolite table, including eight cereal samples in duplicates and 89 identified metabolites. PC1 *versus* PC2 scores plot of the PCA model (Figure 3A) show a clear separation of four different cereals explaining more than 60% variation of the data. The loadings plot of the corresponding model (Figure 3B) demonstrates a large spread of the 89 metabolites and revealed no clear groupings of metabolites classes. However, major part of the benzoic acid derived phenolics such as 3,5-dihydroxybenzoic, 3,4-dihydroxybenzoic and 3,4,5-trihydroxybenzoic acids are grouped on the upper left part of the loadings plot showing greater abundance in barley compared to the other cereals. In contrast to this, cinnamic acid derived phenolics such as ferulic, sinapinic and syringic acids are located on the bottom right corner showing greater concentrations in rye and wheat. Phenolics such as caffeic and 4-hydroxybenzoic acids have the highest concentrations in oat and significantly contribute to its separation from other cereals. However, detailed variations of phenolics and organic acids within and between cereal cultivars require a closer investigation of the data. In the following section, univariate comparisons of some metabolites are represented and the findings are compared to previous results reported in the literature.

### 3.3. Variation of Phenolics and Organic Acids in Cereals

**Figure 4:**
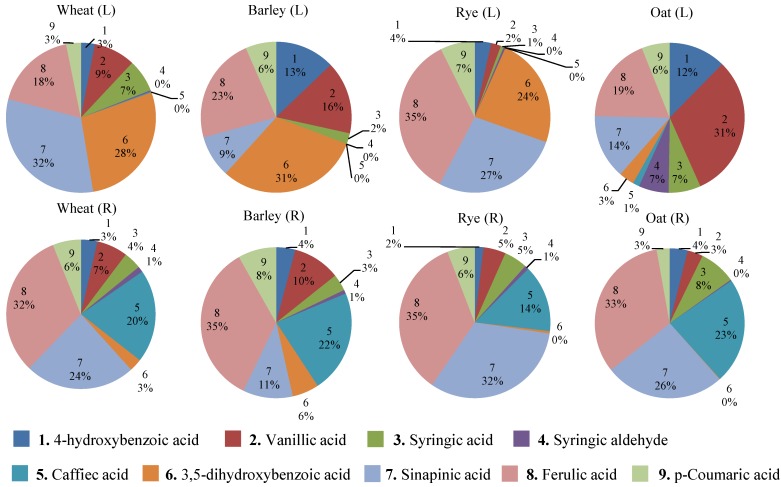
Comparison of relative percentages of the nine most abundant phenolic acids of cereals reported in the literature (L) with the results of the current study (R). In literature the following genotypes were studied: winter wheat (*Triticum aestivum* var. *aestivum*) [18], Dicktoo barley (USA) [21], Grandrieu rye (France) [23] and Bajka oat (Poland) [22].

Phenolic acid composition of wheat, barley, rye and oat were compared to previously reported data [18,21,22,23]. Figure 4 shows relative percentages of the nine most abundant, free and conjugated phenolic acids of cereals reported in previous studies and makes comparisons with the data obtained in the current study. In previous studies, the phenolic acids of cereals were extracted using 80% ethanol followed by hydrolysis of conjugated phenolics in 2 M sodium hydroxide solution and analyzed by LC-DAD. In the current study, free and conjugated phenolics were extracted using 85% methanol, hydrolyzed in 2 M solution of hydrochloric acid followed by GC-MS analysis and PARAFAC2 based data processing. These two methodologies in phenolic profiling of cereals result in several apparent compositional differences. However, it should be underlined that the compared cereal genotypes are different in the two studies and the goal of this study is not a comprehensive comparison of phenolics of cereal varieties, but to demonstrate the power of the standardized cereal metabolomics protocol developed.

Nine major phenolics of the cereals investigated in this study were compared with winter wheat (*Triticum aestivum* var. *aestivum*) [18], Dicktoo barley (USA) [21], Grandrieu rye (France) [23] and Bajka oat (Poland) [22] varieties (Figure 4). Figure 4 shows that the relative concentrations of caffeic acid consistently increased (14%–23%) in all cereal cultivars compared to the previous studies where its abundance was below 1%. Similarly, for wheat, barley and oat, concentrations of ferulic acid increased from approximately 20% to 33%, while the comparison is more consistent for the two rye varieties. These results suggest that in grains, a significant amount of caffeic and ferulic acids are present in conjugated forms that cannot be cleaved by alkaline hydrolysis. Thus, the most abundant phenolic acids in previous cereal metabolomics studies were ferulic, sinapinic and 3,5-dihydroxybenzoic acids, while in this study, ferulic, sinapinic and caffeic acids were the most abundant ones.

**Figure 5:**
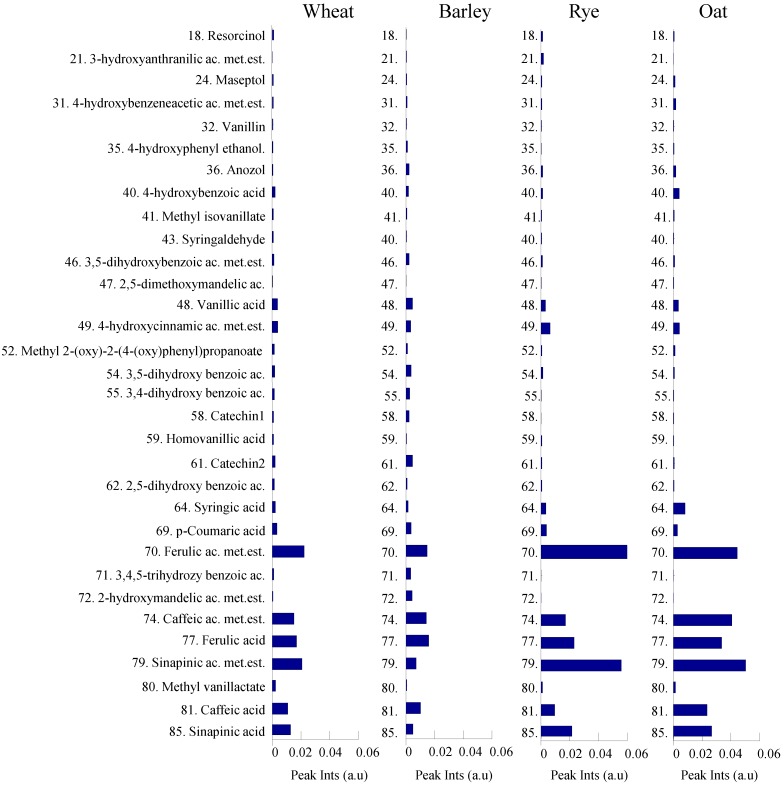
Relative concentrations of 32 phenolics detected from wheat, barley, rye and oat. Metabolites are numbered according to the Table 1.

**Figure 6:**
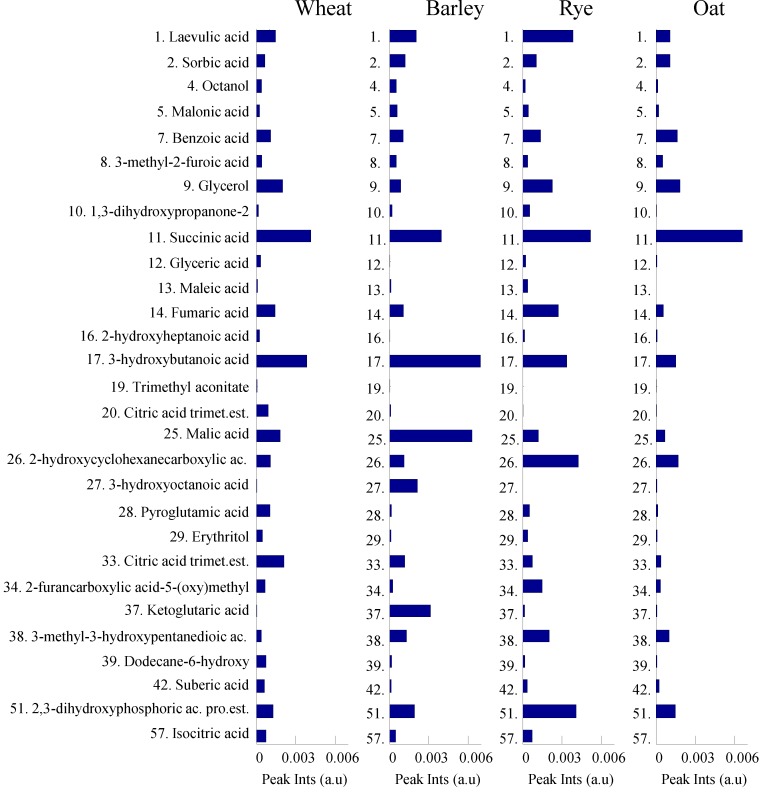
Relative concentrations of 29 organic acids/alcohols detected from wheat, barley, rye and oat. Metabolites are numbered according to the Table 1.

Figure 5 and Figure 6 demonstrate relative concentrations of phenolics and organic acids/alcohols of wheat, barley, rye and oat genotypes investigated in this study. Figure 5 show that ferulic, caffeic and sinapic acids and their methyl esters are the most abundant metabolites among all other phenolics in the cereal samples. Moreover, the relative concentrations of the most abundant phenolics are found to be up to three times greater in rye and oat than in wheat and barley. Succinic and 3-hydroxybutanoic acids were the most abundant metabolites among all organic acids detected in the four different cereals (Figure 5). Relative concentrations of fumaric and 2-hydroxycyclohexanecarboxylic acids were significantly higher in rye, while concentrations of malic and ketoglutaric acids were highest in barley.

## 4. Conclusions

This paper outlines and demonstrates an optimized, relatively unbiased, comprehensive and high-throughput metabolomic profiling of whole-grain cereals based on new technologies developed within GC-MS metabolomics and chemometrics. A metabolite extraction protocol optimized towards phenolics and organic acids of whole-grains, and an unbiased and high-throughput protocol, was developed that allow processing of up to 60 samples per day. The hydrochloric acid based hydrolysis allowed extraction of all major cereal phenolics, free and conjugated, and enabled the detection of 32 phenolic and 30 organic acids from 50 mg of flour. A novel trimethylsilylation method based on TMSCN allowed the detection of up to 300 metabolites from the GC-MS profiles. The multi-way decomposition method PARAFAC2 facilitated deconvolution of overlapping, retention time shifted and low s/n ratio peaks with high precision and in a semi-automated manner. The resolved mass spectra of deconvoluted peaks allowed the identification of 89 metabolites using NIST and Golm metabolite databases. Multivariate and univariate analysis of phenolic profiles of cereals revealed that ferulic, caffeic and sinapinic acids and their esters were the main phenolics of whole-grain samples across the four cereals studied. Rye and oat showed higher concentrations of the most abundant phenolics acids compared to wheat and barley. Comparison of the relative concentrations of the nine most abundant phenolics of cereals with previously reported data showed that the acidic hydrolysis significantly improved detection of caffeic acid. However, metabolite profiles of cereals highly depend on several factors such as genotype, growth conditions, harvest time and storage. Thus, essential secondary metabolite profile comparisons of different cereals as well as different varieties require a strictly controlled experimental design. This paper has demonstrated a new methodology that is ready to be applied in a larger metabolomic profiling studies that may reveal biological information related to phenolic and organic acids of whole-grain cereals. Moreover, the protocol developed can easily be modified for polar metabolite fractions, including mono- and di-saccharides and amino acids, of cereals by altering metabolite extraction method and the additional of a methoximation step in GC-MS derivatization.
